# Premalignant Changes in the Bronchial Epithelium Are Prognostic Factors of Distant Metastasis in Non-Small Cell Lung Cancer Patients

**DOI:** 10.3389/fonc.2021.771802

**Published:** 2021-11-10

**Authors:** Olga V. Pankova, Liubov A. Tashireva, Evgeny O. Rodionov, Sergey V. Miller, Sergey A. Tuzikov, Dmitry S. Pismenny, Tatiana S. Gerashchenko, Marina V. Zavyalova, Sergey V. Vtorushin, Evgeny V. Denisov, Vladimir M. Perelmuter

**Affiliations:** ^1^ Cancer Research Institute, Tomsk National Research Medical Center, Tomsk, Russia; ^2^ Department of Pathological Anatomy, Siberian State Medical University State Medical University, Tomsk, Russia; ^3^ Department of Organic Chemistry, Tomsk State University, Tomsk, Russia

**Keywords:** non-small cell lung cancer (NSCLC), bronchial lesion, distant metastasis, metastasis-free survival, basal cell hyperplasia, squamous cell metaplasia

## Abstract

**Background:**

The study assessed the possibility of dividing patients into groups based on the assessment of morphological changes in the epithelium of small-caliber bronchi located near the primary tumor in order to predict high and low risks of distant metastasis of non-small cell lung cancer.

**Methods:**

In 171 patients with non-small cell lung cancer (T_1-4_N_0-3_M_0_) in small-caliber bronchi taken at a distance of 3–5 cm from the tumor, various variants of morphological changes in the bronchial epithelium (basal cell hyperplasia (BCH), squamous cell metaplasia (SM), and dysplasia (D)) were assessed. Long-term results of treatment, namely, distant metastasis, were assessed after 2 and 5 years.

**Results:**

During the follow-up period, distant metastases were found in 35.1% (60/171) of patients. Most often, they were observed in patients of the high-risk group: BCH+SM−D− (51.6%, 40/95) and BCH−SM+D+ (54.4%, 6/11). Less often, distant metastases were observed in low-risk group patients: BCH+SM+D− (6.7%, 3/45) and BCH−SM−D− (10.0%, 2/20). Tumor size, grade, and stage were significant predictors of metastasis only in the high-risk group. The 5-year metastasis-free survival was better in the low-risk group of distant metastases.

**Conclusions:**

Isolated BCH or dysplasia in small bronchi distant from foci of tumor is associated with a high-risk distant metastasis and less 5-year metastasis-free survival.

## Introduction

The most common cause of cancer death in 2020 was lung cancer (1). High mortality is associated with the progression of the tumor process. Therefore, the search for various molecular biological markers involved in the mechanisms of distant metastasis in non-small cell lung cancer (NSCLC) remains relevant. The identification of patients with a high risk of tumor progression can be used to adequately prescribe adjuvant chemotherapy (AC) and to adjust its regimen in order to minimize adverse effects.

The most important factors associated with progression of NSCLC and predicting survival are tumor stage, histologic structure, grade, and biological aggressiveness (2–5). However, these factors are not always effective in predicting the outcome of the tumor process. Our earlier study showed that different variants of the combination of morphological changes in the epithelium of small bronchi [basal cell hyperplasia (BCH), squamous cell metaplasia (SM), and dysplasia (D)], distant from foci of squamous cell carcinoma and lung adenocarcinoma, are associated with recurrence. The combination of BCH and SM is associated with high risk of recurrence of NSCLC regardless of the histologic type of tumor and neoadjuvant chemotherapy (NAC) (6, 7). In this study, we considered the association of different variants of morphological changes of the respiratory epithelium of small bronchi adjacent to the tumor as risk factors for distant metastasis.

## Methods

### Patients

The study enrolled 171 patients with NSCLC (squamous cell carcinoma and adenocarcinoma, T_1-4_N_0-3_M_0_) who were treated in the Cancer Research Institute, Tomsk NRMC, between 2005 and 2011. Patients were excluded if they were refused surgery and had an Eastern Cooperative Oncology Group (ECOG)/WHO performance score >2, small-cell lung cancer, associated severe diseases, and cardiovascular and pulmonary decompensation. Metastatic involvement was identified from the Local Cancer Register. The study was approved by the Institutional Review Board (IRB) (December 10, 2012; the number of approvals is 16).

**Figure 1:**
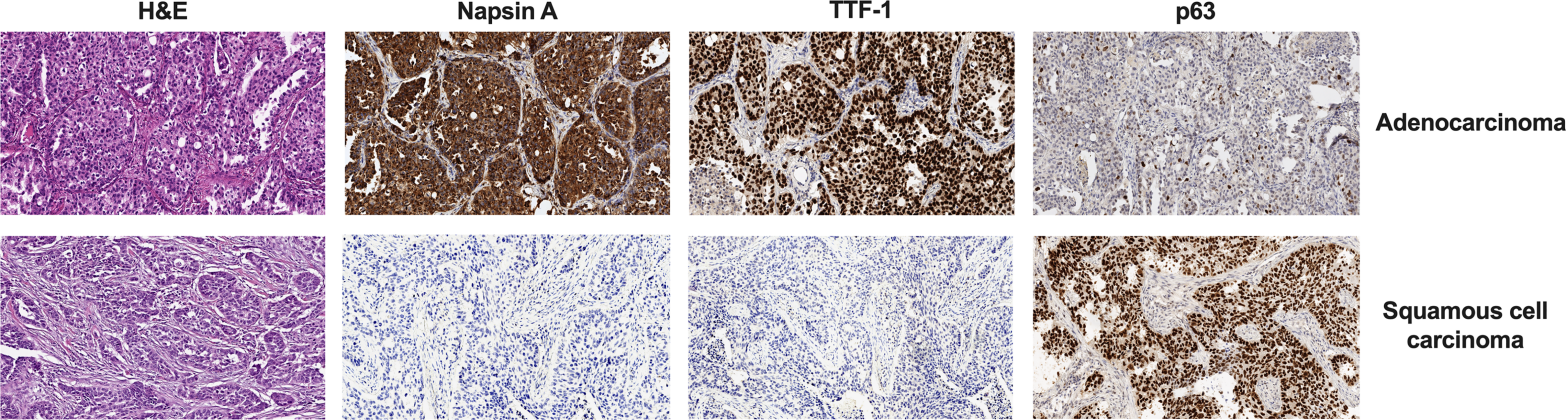
Difference in H&E and IHC staining of two histologic types of NSCLC. Magnification, ×100. IHC, immunohistochemistry; NSCLC, non-small cell lung cancer.

The histologic diagnosis of lung cancer was made according to the International Association for the Study of Lung Cancer/American Thoracic Society/European Respiratory Society (IASLC/ATS/ERS) lung adenocarcinoma classification (8) and the WHO criteria (9) and was confirmed by immunohistochemistry using a panel of antibodies: TTF-1 (clone 8G7G3/1, Dako), Napsin A (Rabbit Polyclonal, Cell Marque), and p63 (Rabbit Polyclonal, Leica) (**Figure 1**).

Cancer stage was determined according to the TNM classification (10). The type of morphological lesions in the bronchial epithelium (BCH, SM, and D) of small bronchi (d = 0.5–2 mm), obtained at a distance of ~3 cm from the tumor edge during surgery, was assessed as described earlier (11).

### Statistical Analysis

The data were analyzed with the statistical software STATISTICA 12 (StatSoft, OK, USA) and GraphPad Prism 9 (GraphPad Software, San Diego, CA, USA). A multivariate logistic regression model was used to calculate odds ratios (OR) for type of bronchial lesions, histologic type, recurrence, type of therapy, gender, smoking status, grade, and stages T and N. p-Values for 2 × 2 tables were obtained by using Fisher’s exact test. Survival was investigated with univariate and multivariate Cox regression models, yielding hazard ratios (HRs). This model adjusted for type of bronchial lesions, histologic type, recurrence, type of therapy, gender, smoking status, grade, and stages T and N. Metastasis-free survival (MFS) was calculated by the Kaplan–Meier method, and differences in survival curves among the groups were evaluated by the log rank test. p < 0.05 was considered statistically significant.

## Results

Over the entire follow-up period, distant metastases occurred in 35.1% (60/171) of patients with NSCLC. The clinical and pathological parameters of NSCLC patients depending on the presence or absence of distant metastasis are presented in **Table 1**.

**Table 1:** Clinicopathologic features of NSCLC patients with or without distant metastases.

Parameter	*Number of patients*, n (%)		p
	MTS+	MTS−	
Gender			
M	55/60 (91.7)	88/111 (79.3)	0.05
F	5/60 (8.3)	23/111 (20.7)
Age	57.1 ± 4.2	58.3 ± 8.6	0.51
Smoking	49/60 (81.7)	79/111 (71.2)	
Non-smoking	11/60 (18.3)	32/111 (28.8)	0.14
Histologic type			
Squamous cell carcinoma	41/60 (68.3)	66/111 (59.5)	0.32
Adenocarcinoma	19/60 (31.7)	45/111 (40.5)
Stage			
I	2/60 (3.3)	12/111 (10.8)	0.07
IIА	2/60 (3.3)	7/111 (6.3)	0.38
IIB	5/60 (8.3)	29/111 (26.1)	0.005
IIIА	40/60 (66.7)	44/111 (39.6)	0.0009
IIIB	11/60 (18.4)	19/111 (17.2)	0.83
Tumor size			
Т_1–2_	9/60 (15.0)	42/111 (37.8)	0.002
Т_3–4_	51/60 (85.0)	69/111 (62.2)
Grade			
1	2/60 (3.3)	8/111 (7.2)	0.27
2	48/60 (80.0)	56/111 (50.5)	0.0003
3	10/60 (16.7)	47/111 (42.3)	0.0011
Nodal status			
Positive	31/60 (51.7)	51/111 (45.9)	0.45
Negative	29/60 (48.3)	60/111 (54.1)
Recurrence			
Yes	3/60 (5.0)	20/111 (18.0)	0.48
No	57/60 (95.0)	91/111 (82.0)	0.02
NAC			
Yes	27/60 (45.0)	63/111 (56.8)	0.152
No	33/60 (55.0)	48/111 (43.2)
IORT			0.172
Yes	9/60 (15.0)	28/111 (25.2)
No	51/60 (55.0)	83/111 (74.8)
NAC effect			
Partial regression	8/27 (29.6)	26/63 (41.3)	0.34
Stabilization	19/27 (70.4)	37/63 (58.7)	0.29
AC			
Yes	31/60 (51.7)	50/111 (45.05)	0.426
No	29/60 (48.3)	61/111 (54.95)
AC regimen			
Vinorelbine/carboplatin	9/60 (15.0)	21/111 (18.9)	0.51
Paclitaxel/carboplatin	12/60 (20.0)	14/111 (12.6)	0.23
Gemcitabine/carboplatin	6/60 (10.0)	13/111 (11.7)	0.69
Irinotecan/carboplatin	2/60 (3.3)	2/111 (1.8)	0.61
Etoposide/cisplatin	2/60 (3.3)	0/111 (0.0)	0.07

Fisher’s exact test was used.

MTS, distant metastasis; NSCLC, non-small cell lung cancer; NAC, neoadjuvant chemotherapy; IORT, intraoperative radiotherapy; AC, adjuvant chemotherapy.

Of those cases that developed metastasis, 81.7% (49/60) had BCH+SM−D−; 10.0% (6/60), BCH−SM+D+; 5.0% (3/60), BCH+SM+D−; and 3.3% (2/60), BCH−SM−D−. Patients with BCH+SM−D− and BCH−SM+D+ had the highest percentage (51.6% (49/95) and 54.5% (6/11), respectively) of metastasis than had BCH+SM+D− [6.7% (3/45)] and BCH−SM−D− [10.0% (2/20)] patients.

Based on these results, we identified two groups of patients: with low (BCH−SM−D− and BCH+SM+D−) and high (BCH+ SM−D− and BCH−SM+D+) risk of distant metastases. We used univariate and multivariate prognostic analyses to assess the prognostic effect of the risk score system based on type of bronchial lesions and clinical and pathologic parameters of NSCLC patients (**Table 2**).

**Table 2:** Univariate and multivariate logistic regression analyses of factors associated with distant metastasis in NSCLC patients.

	Univariate logistic regression analysis		Multivariate logistic regression analysis of variables significant after univariate analysis		Multivariate logistic regression analysis with all variables	
	OR (95% CI)	p-Value	OR (95% CI)	p-Value	OR (95% CI)	p-Value
Risk group	12.94 (4.81–34.78)	0.0000	15.63 (5.38–45.41)	0.0000	34.88 (7.87–154.60)	0.0000
*Low vs. high*						
Histologic type	0.62 (0.32–1.18)	0.1473	–	–	0.76 (0.25–2.28)	0.6370
*Squamous cell carcinoma vs. adenocarcinoma*						
Recurrence	2.89 (0.93–8.93)	0.0651	–	–	0.37 (0.04–2.87)	0.3470
*No vs. yes*						
NAC	1.54 (0.82–2.90)	0.1761	–	–	1.04 (0.30–3.58)	0.9489
*No vs. yes*						
IORT	1.60 (0.74–3.43)	0.2259	–	–	1.38 (0.50–3.80)	0.5288
*No vs. yes*						
Gender	0.36 (0.13–1.01)	0.0537	–	–	0.34 (0.04–2.56)	0.2966
*Male vs. female*						
Tumor size	3.44 (1.54–7.72)	0.0025	6.05 (2.29–15.96)	0.0002	11.65 (3.43–39.47)	0.0001
*1–2 vs. 3–4*						
Nodal status	0.79 (0.42–1.49)	0.4751	–	–	0.63 (0.21–1.86)	0.4069
*Negative vs. positive*						
AC	1.53 (0.81–2.88)	0.1873	–	–	1.76 (0.65–4.75)	0.2623
*No vs. yes*						
Smoking status	0.55 (0.25–1.19)	0.1341	–	–	0.84 (0.27–2.55)	0.7650
*No vs. yes*						
Grade	3.67 (1.68–7.98)	0.0010	7.38 (2.90–18.73)	0.0000	15.52 (4.57–52.65)	0.0000
*1–2 vs. 3*						
Stage group	5.13 (2.23–11.82)	0.0001	11.89 (4.23–33.39)	0.0000	8.23 (2.87–22.59)	0.0001
I–II vs. III						

NSCLC, non-small cell lung cancer; NAC, neoadjuvant chemotherapy; IORT, intraoperative radiotherapy; AC, adjuvant chemotherapy.

In the univariate logistic regression analysis, the high-risk group (OR = 12.9; 95% CI = 4.8–34.7, p < 0.001), T3-4 (OR = 3.4; 95% CI = 1.5–7.7, p = 0.002), grade 3 (OR = 3.6; 95% CI = 1.6–7.9, p = 0.001), and stage III (OR = 5.1; 95% CI = 2.2–11.8, p = 0.0001) were significantly associated with higher risks of distant metastasis. The ORs of histologic type, nodal status, recurrence, NAC, intraoperative radiotherapy (IORT), gender, smoking status, and AC (p > 0.05) were insignificant in the univariate logistic regression analysis. The multivariate logistic regression models (all variables and variables significant after univariate analysis) confirmed that the high-risk group, T3-4, grade 3, and stage III (OR > 1; p < 0.001) were still significantly associated with higher risks of metastasis. As a result, based on OR, being a high-risk group was the most influential risk factor for distant metastasis of NSCLC.

**Figure 2:**
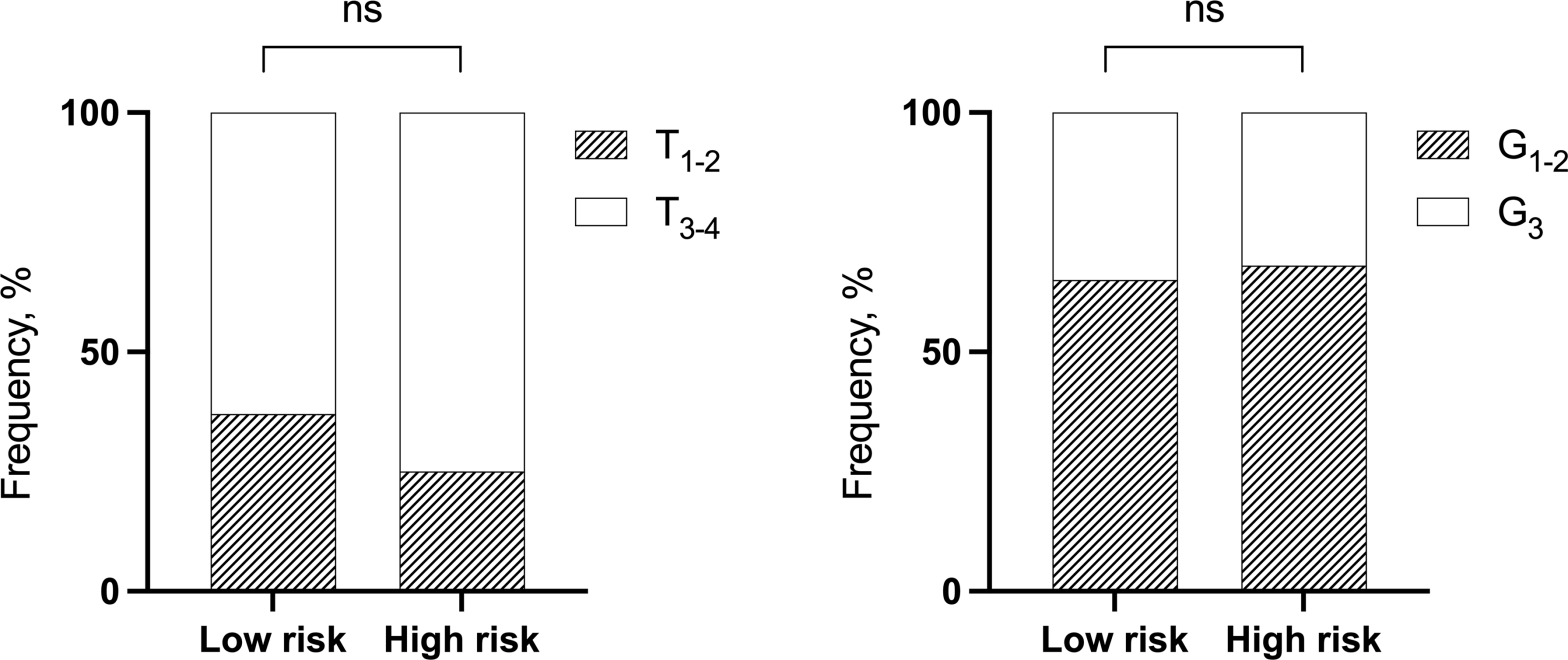
The distribution of NSCLC patients by T and grade depending on low- and high-risk groups of distant metastases. Fisher’s exact test. NSCLC, non-small cell lung cancer; ns, not significant.

The frequency of the stage T at diagnosis and the grade between the low- and high-risk groups were not significant (Fisher’s exact test, p > 0.05) (**Figure 2**).

This, as well as no correlation between stage T and grade (r^2^ = 0.3448, p = 0.6998), may indicate the independence of three factors in the distant metastasis prognosis. The results presented in **Table 3** allow us to compare the significance of three factors (risk groups, T, and grade) to determine the rate of developing distant metastases in NSCLC.

**Table 3:** The frequency of distant metastases depending on the cancer stage and the grade, separately for the low- and high-risk in NSCLC patients.

		The rate of distant metastases, n (%)	
		Low-risk group	High-risk group
		1	2
Т_1–2_	a	1/24 (4.16)	8/27 (29.6)
			р_a–b_ = 0.026
Т_3–4_	b	4/41 (9.75)	47/79 (59.4)
		р_a–b_ = 0.6438	р_1–2_ = 0.0000
			р_a–b_ = 0.0132
Grade 1–2	c	0/23 (0)	10/34 (29.4)
			р_1–2_ = 0.0037
Grade 3	d	5/42 (11.9)	45/72 (62.5)
		р_c–d_ = 0.1521	р_1–2_ = 0.0000
			р_c–d_ = 0.0018

Fisher’s exact test was used.

There is every reason to believe that the assignment of patients to high- and low-risk groups by the risk score system based on type of bronchial lesions is the most significant and independent prognostic factor of distant metastasis. Moreover, it is acceptable to believe that T3-4 and grade 3 are unfavorable factors only in the high-risk group of distant metastases.

**Figure 3:**
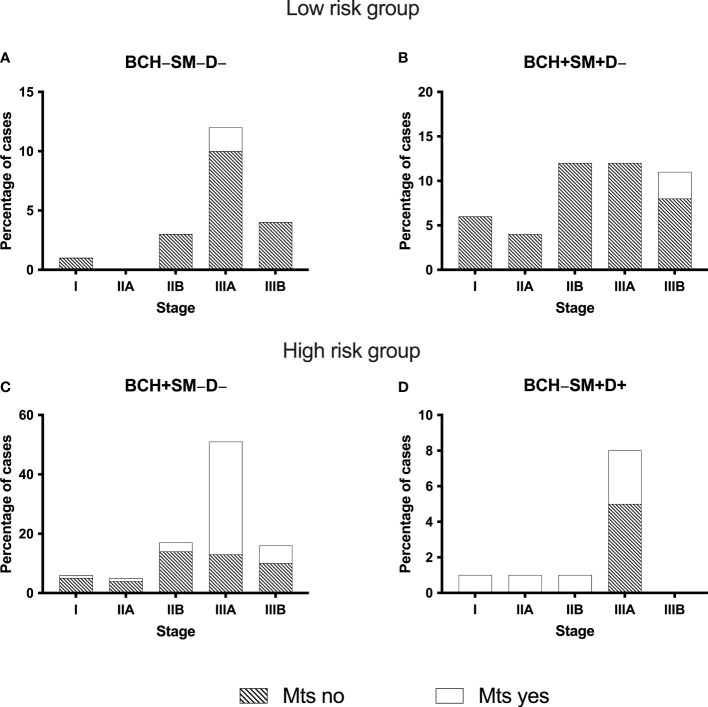
The frequency of distant metastases in NSCLC patients with different bronchial lesions **(A–D)** depending on the cancer stage. BCH, basal cell hyperplasia; SM, squamous metaplasia; D, dysplasia; Mts, distant metastases; NSCLC, non-small cell lung cancer.

We described the frequency of metastases depending on the cancer stage and the grade, separately for the low- and high-risk groups (**Figure 3**).

The evaluation of the relationship of distant metastasis of NSCLC with the cancer stage showed that in BCH−SM−D− group single distant metastases occurred only in patients with stage IIIA, 16.7% (2/12) (**Figure 3A**). In another low-risk group (BCH+SM+D−), metastases were in 11.1% (1/9) of patients with stage IIB, in 8.3% (1/12) of patients with IIIA, and in 9.09% (1/11) of patients with stage IIIB (**Figure 3B**).

There were no metastases in the low-risk group of distant metastases in I–II stage during the observation period, while in the high-risk group of distant metastases (BCH+SM−D− and BCH−SM+D+), metastases occurred at any cancer stage (**Figures 3C, D**). From **Table 4**, it follows that only the high-risk group of distant metastases is associated with the cancer stage (p = 0.0028).

**Table 4:** The frequency of distant metastases in groups of high and low risk of metastasis, depending on the cancer in NSCLC patients.

Stage		The frequency of distant metastases, n (%)	
		Low-risk group	High-risk group
I	a	0/7 (0)	2/7 (28.57)
			p = 0.4615
IIA	b	0/4 (0)	2/6 (33.33)
			p = 0.4667
IIB	c	0/15 (0)	4/18 (22.22)
			p = 0.1081
IIIA	d	2/24 (8.33)	41/59 (69.49)
			p = 0.0000
IIIB	e	3/15 (20.00)	6/16 (37.50)
			p = 0.4331
p-Value		p_abc–de_ = 0.0777	p_abc–de_ = 0.0028

Fisher’s exact test was used.

The comparison of the rates of metastasis in cases with stage IIIA shows that at the same stage, the frequency of metastasis in the high-risk group is 20 times higher than in the low-risk group.

### Survival Analyses

We explored the potential prognostic factors in NSCLC patients using univariate and multivariate Cox regression analyses. None of the investigated parameters in univariate and multivariate Cox analyses influenced 2-year MFS in NSCLC patients (**Table 5**).

**Table 5:** Univariate and multivariate Cox regression analyses of prognostic factors for 2-year metastasis-free survival in NSCLC patients.

Features	Level	Metastasis-free survival			
		Univariate		Multivariate	
		Hazard ratio (95% CI)	p-Value	Hazard ratio (95% CI)	p-Value
Risk group	*Low*	Ref		Ref	
	*High*	0.83 (0.58–1.19)	0.3195	0.88 (0.54–1.29)	0.5845
Type of bronchial lesions	*BCH−SM−D−*	Ref		Ref	
	*BCH+SM−D−*	1.02 (0.53–1.99)	0.9364	1.00 (0.50–1.98)	0.9149
	*BCH+SM+D−*	0.98 (0.57–1.71)	0.7452	0.96 (0.49–1.88)	0.8730
	*BCH−SM+D+*	0.85 (0.38–1.89)	0.6993	0.85 (0.36–2.01)	0.7606
Histologic type	*Squamous cell carcinoma*	Ref		Ref	
	*Adenocarcinoma*	1.04 (0.71–1.52)	0.8051	0.99 (0.65–1.50)	0.9855
Recurrence	*No*	Ref		Ref	
	*Yes*	0.63 (0.39–1.01)	0.0529	0.97 (0.52–1.80)	0.9312
Type of therapy	*NAC−IORT−*	Ref		Ref	
	*NAC+IORT−*	1.10 (0.76–1.59)	0.6491	1.04 (0.64–1.68)	0.9407
	*NAC+IORT+*	1.03 (0.69–1.54)	0.9368	1.12 (0.75–1.66)	0.6087
Gender	*Male*	Ref		Ref	
	*Female*	1.27 (0.79–2.04)	0.3120	1.06 (0.62–1.85)	0.8186
Tumor size	*T1–2*	Ref		Ref	
	*T3–4*	1.07 (0.73–1.57)	0.7145	0.99 (0.67–1.47)	0.9956
Nodal status	*Negative*	Ref		Ref	
	*Positive*	0.90 (0.63–1.30)	0.5956	1.01 (0.66–1.55)	0.9328
AC	*No*	Ref		Ref	
	*Yes*	0.91 (0.64–1.31)	0.6479	1.05 (0.71–1.55)	0.7795
Smoking status	*Smoking*	Ref		Ref	
	*Non-smoking*	1.03 (0.69–1.53)	0.8750	0.99 (0.66–1.46)	0.9629
Grade	*1–2*	Ref		Ref	
	*3*	1.14 (0.78–1.64)	0.4839	0.88 (0.60–1.29)	0.5350

NSCLC, non-small cell lung cancer; BCH, basal cell hyperplasia; SM, squamous cell metaplasia; D, dysplasia; NAC, neoadjuvant chemotherapy; IORT, intraoperative radiotherapy; AC, adjuvant chemotherapy.

As shown in **Table 6**, the univariate Cox regression analysis revealed that the BCH+SM−D− type of bronchial lesions was significantly associated with poor 5-year MFS in patients with NSCLC.

**Table 6:** Univariate and multivariate Cox regression analyses of prognostic factors for 5-year metastasis-free survival in NSCLC patients.

Features	Level	Metastasis-free survival			
		Univariate		Multivariate	
		Hazard ratio (95% CI)	p-Value	Hazard ratio (95% CI)	p-Value
Risk group	*Low*	Ref		Ref	
	*High*	0.78 (0.50–1.21)	0.2745	0.67 (0.46–1.14)	0.1543
Type of bronchial lesions	*BCH−SM−D−*	Ref		Ref	
	*BCH+SM−D−*	1.56 (1.09–2.72)	0.0442	2.20 (1.05–4.58)	0.0209
	*BCH+SM+D−*	1.18 (0.58–2.38)	0.7125	1.50 (0.69–3.25)	0.6981
	*BCH−SM+D+*	1.11 (0.50–2.48)	0.8219	1.03 (0.43–2.47)	0.4496
Histologic type	*Squamous cell carcinoma*	Ref		Ref	
	*Adenocarcinoma*	0.85 (0.53–1.38)	0.5343	0.92 (0.56–1.49)	0.7372
Recurrence	*No*	Ref		Ref	
	*Yes*	0.72 (0.42–1.26)	0.2606	1.06 (0.55–2.03)	0.8506
Type of therapy	*NAC−IORT−*	Ref		Ref	
	*NAC+IORT−*	0.78 (0.51–1.19)	0.8574	0.52 (0.28–1.01)	0.0704
	*NAC+IORT+*	0.65 (0.41–1.03)	0.1710	0.72 (0.45–1.51)	0.9939
Gender	*Male*	Ref		Ref	
	*Female*	1.50 (0.81–2.79)	0.1948	1.40 (0.75–2.61)	0.2871
Tumor size	*T1–2*	Ref		Ref	
	*T3–4*	0.89 (0.57–1.38)	0.6138	0.98 (0.62–1.56)	0.9638
Nodal status	*Negative*	Ref		Ref	
	*Positive*	1.02 (0.64–1.62)	0.9134	0.86 (0.52–1.43)	0.5821
AC	*No*	Ref		Ref	
	*Yes*	0.94 (0.61–1.45)	0.7930	0.98 (0.63–1.55)	0.9638
Smoking status	*Smoking*	Ref		Ref	
	*Non-smoking*	0.97 (0.62–1.51)	0.9052	1.02 (0.66–1.58)	0.9183
Grade	*1–2*	Ref		Ref	
	*3*	1.24 (0.79–1.94)	0.3300	0.95 (0.59–1.51)	0.8390

NSCLC, non-small cell lung cancer; BCH, basal cell hyperplasia; SM, squamous cell metaplasia; D, dysplasia; NAC, neoadjuvant chemotherapy; IORT, intraoperative radiotherapy; AC, adjuvant chemotherapy.

**Figure 4:**
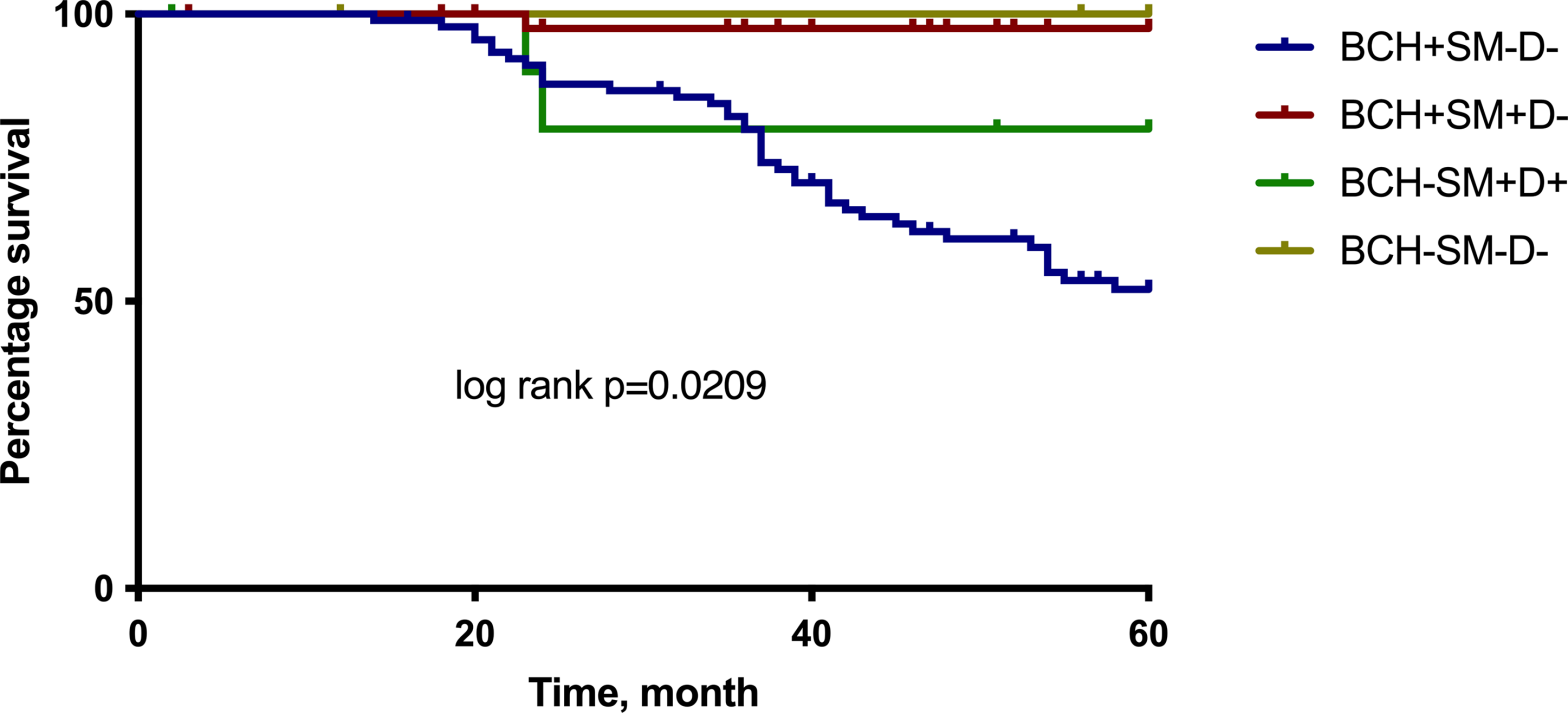
The 5-year MFS for NSCLC patients according to the different types of bronchial lesions. Log rank test. MFS, metastasis-free survival.

The multivariate Cox regression analysis showed that the BCH+SM−D− type of bronchial lesions was an independent prognostic factor for the 5-year MFS. The Kaplan–Meier plots indicated that NSCLC patients with BCH+SM−D− exerted significantly worse survival than the patients with other type of bronchial lesions (p < 0.05; **Figure 4**).

The survival rates of NSCLC patients with different types of bronchial lesions who were alive for 5 years was 52.0% at BCH+SM−D−, 97.5% at BCH+SM+D−, 80.0% at BCH−SM+D+, and 100.0% at BCH−SM−D−.

**Figure 5:**
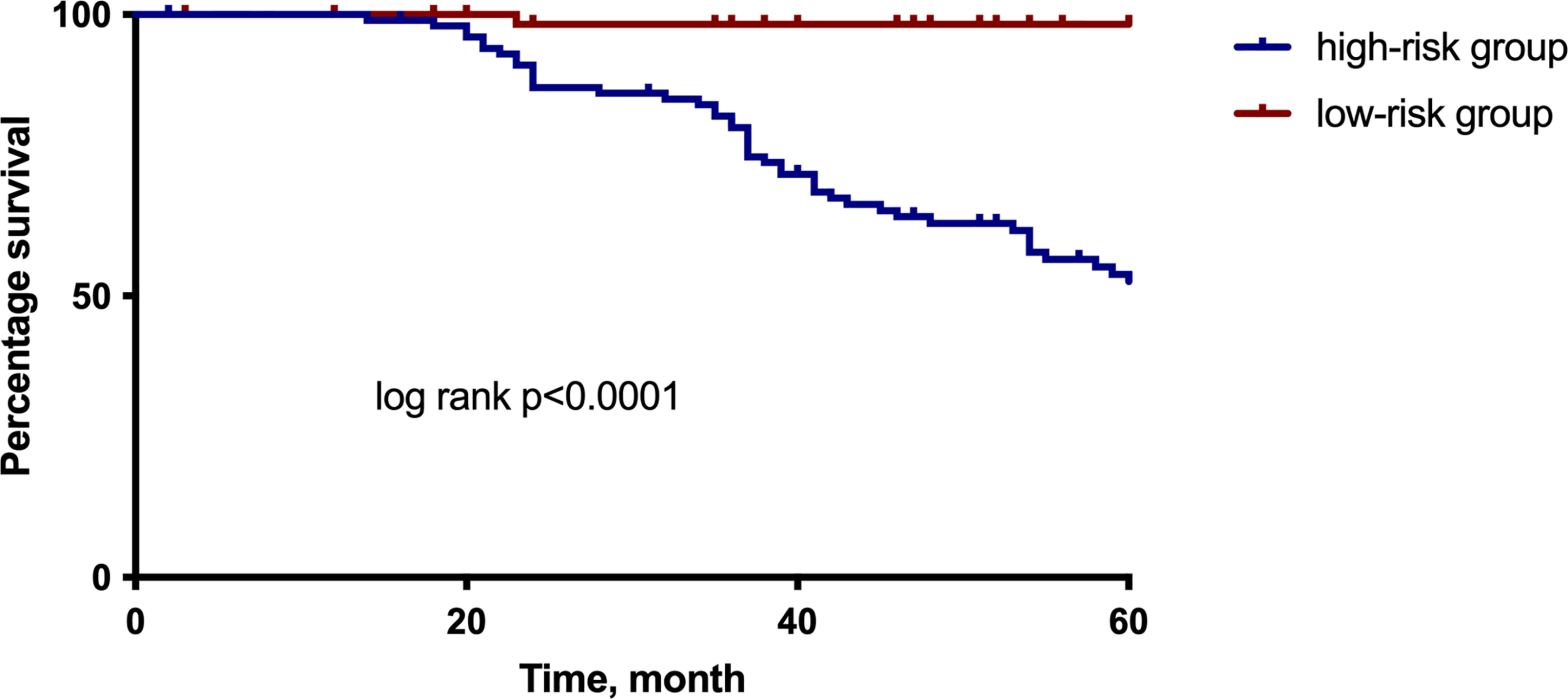
The 5-year MFS for NSCLC patients according to the group of low and high risk of distant metastasis. Log rank test. MFS, metastasis-free survival.

Even more clearly, the association between the type of bronchial lesions and the frequency of distant metastasis was demonstrated when evaluating the curves of 5-year MFS in the low- and high-risk groups (**Figure 5**).

The survival rates of the high-risk and low-risk groups of patients who were either diagnosed with NSCLC or who were alive at 5 years were 52.5% and 98.2%, respectively.

## Discussion

The results of the study indicate that the state of the epithelium in the small bronchi distant from the tumor can be considered as a factor that can be used to divide patients with NSCLC into groups of low and high risk of distant metastasis. The absence of changes in the epithelium (BCH−SM−D−) or the combination of BCH with squamous metaplasia (BCH+SM+D−) is associated with a low frequency of metastasis (10% or 6.7%, respectively). A high risk of distant metastases is associated with the isolated BCH (BCH+SM−D−) and SM combined with D of the respiratory epithelium (BCH−SM+D+).

Metastases in these groups were found in 51.6% and 54.5% of cases. The significance of the type of morphological premalignant changes in the epithelium of small bronchi is a predictor of the incidence of distant metastases, and the time of their clinical manifestation is also confirmed by the results of the Cox regression analyses. In the low-risk group of distant metastases, 5-year MFS was higher. It is noteworthy that the study demonstrated the significance of generally recognized factors in predicting distant metastasis: tumor size, grade, and stage of the process. However, an important innovation lies in the fact that these factors have a significant prognostic ability precisely in the high-risk group, stratified by type of bronchial lesions.

It is known that microenvironment determines the invasiveness and ability of cells to intravasate, which is the first step in the metastatic process. This view explains the probability of a complex chain of cause-and-effect relationships between parenchymal–stromal relationships in small bronchi located near the tumor and the risk of distant metastasis of NSCLC.

The results of the study suggest that the variant of a combination of different types of morphological and molecular changes in the bronchial epithelium under conditions of chronic inflammation in the bronchi (in chronic bronchitis or NSCLC) is a stable condition reflecting the constitutive features of stromal–parenchymal relationships during inflammation and the divergent nature of the progression of precancerous changes in the bronchial epithelium.

Previously, we analyzed the expression profiles of the BCH, SM, and D genes in small bronchi near the primary tumor in NSCLC. It was found that isolated BCH in the high-risk group of distant metastasis differs from the BCH combined with SM in the low-risk group of metastases (BCH+SM+D−) by the expression of immune response genes. Increased expression of genes for regulation of the cell cycle and downregulation of genes for assembly of cilia of the epithelium distinguish SM combined with D in the second high-risk group (BCH−SM+D+) from SM, which is combined with BCH in the group of low risk of metastasis (BCH+SM+D−). Finally, in the epithelium with dysplastic changes, overexpression of genes regulating cell division and insufficient expression of genes regulating inflammation are noted (7). The described differences in gene expression suggest variability in epithelial–stromal relationships, including the nature of inflammation and the response of the epithelium to inflammation-associated cytokines, with different combinations of morphological changes in the respiratory epithelium. Because of this, it can be expected that different variants of epithelial–stromal relations in small bronchi adjacent to the tumor, which may have a constitutive nature, may be associated with different variants of parenchymal–stromal relations in carcinomas, which substantially determine the risk of distant metastasis.

Unfortunately, prior to this study, there was no concept of morphological changes in the epithelium of small bronchi as a factor associated with distant metastasis, and their prognostic significance in patients with NSCLC. Numerous studies are aimed at characterizing different types of changes in the epithelium, their order, reversibility, and, most importantly, the significance in the occurrence of carcinomas. Particular attention, of course, is paid to D as a precancer process. Overall, there are no studies about the mechanisms of the association discussed in this paper. We can only suggest a possible mechanism underlying the association of variants of morphological changes in the bronchi adjacent to the tumor with the risk of distant metastasis in lung cancer. It is assumed that the mechanism of this relationship is due to the genetic determination of immune-inflammatory reactions in the development of chronic bronchitis and the types of epithelial–stromal interactions associated with them. Shortly, various combinations of morphological changes in the bronchi reflect the constitutive features of parenchymal–stromal relations, which are formed under conditions of chronic inflammation in the bronchial wall.

A possible chain of events could be presented as follows: 1) constitutively isolated variants of the development of the immune-inflammatory reaction in the bronchial mucosa determine the dominant spectrum of cytokines affecting the epithelium. 2) Depending on the cytokines affecting the epithelium of the bronchi and/or the response of the epithelium to the action of cytokines, different morphological changes and their combinations develop in the bronchi. 3) Different variants of morphological changes in small bronchi in NSCLC are markers of discrete variants of epithelial–stromal relations during chronic inflammation and can be a criterion for discriminating patients by group. 4) The invasive phenotype of primary tumor cells and their ability to intravasate are largely formed under the influence of the microenvironment. 5) In the tumor microenvironment, immuno-inflammatory reactions almost are observed (“non-healing wound”). 6) The nature of inflammation and the spectrum of cytokines in the tumor microenvironment are determined not only by the tumor specificity but also by the reflection of the constitutive features of the development of immune-inflammatory reactions. 7) Constitutive features of immune-inflammatory reactions and epithelial–stromal relations in the wall of bronchi adjacent to the tumor reflect significant manifestations of immune-inflammatory reactions in the tumor microenvironment, including their ability to increase the invasive and intravasation potential and the ability to metastasize. 8) The morphological type of precancerous changes in the bronchial epithelium of the bronchi adjacent to the tumor is a prognostic sign of the risk of metastasis.

## Data Availability Statement

The raw data supporting the conclusions of this article will be made available by the authors, without undue reservation.

## Ethics Statement

The studies involving human participants were reviewed and approved by local ethics committee of the Cancer Research Institute, Tomsk NRMC, on December 10, 2012 (the number of approval is 16). The patients/participants provided their written informed consent to participate in this study.

## Author Contributions

Conceptualization: OP and VP. Methodology: OP and MZ. Investigation: OP, LT, TG, and DP. Provided patients’ data: ER, ST, and SM. Writing—original draft preparation: OP. Writing—review and editing: LT, VP, and SV. Project administration: OP. Funding acquisition: ED. All authors contributed to the article and approved the submitted version.

## Funding

This work was supported by the Russian Science Foundation [grant number 20-75-10060].

## Conflict of Interest

The authors declare that the research was conducted in the absence of any commercial or financial relationships that could be construed as a potential conflict of interest.

## Publisher’s Note

All claims expressed in this article are solely those of the authors and do not necessarily represent those of their affiliated organizations, or those of the publisher, the editors and the reviewers. Any product that may be evaluated in this article, or claim that may be made by its manufacturer, is not guaranteed or endorsed by the publisher.
